# Glycine Relieves Intestinal Injury by Maintaining mTOR Signaling and Suppressing AMPK, TLR4, and NOD Signaling in Weaned Piglets after Lipopolysaccharide Challenge

**DOI:** 10.3390/ijms19071980

**Published:** 2018-07-06

**Authors:** Xiao Xu, Xiuying Wang, Huanting Wu, Huiling Zhu, Congcong Liu, Yongqing Hou, Bing Dai, Xiuting Liu, Yulan Liu

**Affiliations:** 1 Hubei Key Laboratory of Animal Nutrition and Feed Science, Hubei Collaborative Innovation Center for Animal Nutrition and Feed Safety, Wuhan Polytechnic University, Wuhan 430023, China; xuxiao200315@163.com (X.X.); xiuyingdk@foxmail.com (X.W.); wht18842@126.com (H.W.); zhuhuiling2004@sina.com (H.Z.); 13545906457@163.com (C.L.); houyq@aliyun.com (Y.H.); 2 Zhe Jiang Goshine Test Technologies Co., Ltd., Hangzhou 310030, China; db@gojue.com (B.D.); lxt@gojue.com (X.L.)

**Keywords:** glycine, inflammatory response, intestine, LPS, weanling piglets

## Abstract

This study was conducted to envaluate whether glycine could alleviate *Escherichia coli* lipopolysaccharide (LPS)-induced intestinal injury by regulating intestinal epithelial energy status, protein synthesis, and inflammatory response via AMPK, mTOR, TLR4, and NOD signaling pathways. A total of 24 weanling piglets were randomly allotted to 1 of 4 treatments: (1) non-challenged control; (2) LPS-challenged control; (3) LPS + 1% glycine; (4) LPS + 2% glycine. After 28 days feeding, piglets were injected intraperitoneally with saline or LPS. The pigs were slaughtered and intestinal samples were collected at 4 h postinjection. The mRNA expression of key genes in these signaling pathways was measured by real-time PCR. The protein abundance was measured by Western blot analysis. Supplementation with glycine increased jejunal villus height/crypt depth ratio. Glycine also increased the jejunal and ileal protein content, RNA/DNA ratio, and jejunal protein/DNA ratio. The activities of citroyl synthetase in ileum, and α-ketoglutarate dehydrogenase complex in jejunum, were increased in the piglets fed diets supplemented with glycine. In addition, glycine decreased the jejunal and ileal phosphorylation of AMPKα, and increased ileal phosphorylation of mTOR. Furthermore, glycine downregulated the mRNA expression of key genes in inflammatory signaling. Meanwhile, glycine increased the mRNA expression of negative regulators of inflammatory signaling. These results indicate that glycine supplementation could improve energy status and protein synthesis by regulating AMPK and mTOR signaling pathways, and relieve inflammation by inhibiting of TLR4 and NOD signaling pathways to alleviate intestinal injury in LPS-challenged piglets.

## 1. Introduction

The intestinal epithelium not only plays a key role in digestion and absorption of nutrients, but also has an important function in preventing pathogen invasion and dissemination of commensals [1]. However, the intestinal epithelial health status, especially in young animals, could be easily injured by many factors, such as inflammation and infection [2]. Inflammation often results in intestinal mucosal damage and dysfunction, which negatively affects animal performance and health [3]. In order to alleviate the inflammation and maintain health and function, the intestine needs a high level of energy and amino acids (AAs) [4,5].

Glycine, whose structure is the simplest of all AAs, is regarded as a conditionally essential AA for young mammals, and a nutritionally essential amino acid for fetal and neonatal development of poultry [6,7]. In addition, glycine is the most abundant AA in the body [8], and it is highly required for neonatal growth and development [7,9]. Recently, some reports showed that glycine could alleviate colitis induced by chemicals, small intestine injury induced by endotoxins, and inhibit overproduction of pro-inflammatory cytokines in rats [10,11,12].

Activation of inflammatory signaling pathways can lead to intestinal damage [13]. Toll-like receptors (TLRs) and nucleotide-binding oligomerization domain proteins (NODs) are important protein families of inflammatory signaling pathways [14,15]. These proteins are expressed in many tissues, including the intestine [16,17,18], and play key roles in induction of inflammatory responses by recognition of pathogen-associated molecular patterns (PAMPs) [16,19]. Interactions of TLRs or NODs with their specific PAMPs trigger downstream signaling events that lead to activation of nuclear factor-κB (NF-κB), which could further induce the expression of genes related to pro-inflammatory cytokines, such as interleukin-1β (IL-1β), IL-6 and tumor necrosis factor-α (TNF-α) [20]. As a consequence, these pro-inflammatory cytokines adjust the host’s defense against invading pathogens. However, the intestine can be easily injured by overproduction of these cytokines, especially TNF-α. Adenosine monophosphate-activated protein kinase (AMPK) is a serine/threonine protein kinase, which widely exists in eukaryotic cells [21]. AMPK can directly mediate metabolic adaptations to a change of energy status [22]. The overproduction of cytokines, such as TNF-α, increases energy consumption which could activate AMPK [23]. The activated AMPK could further inhibit the mammalian target of rapamycin (mTOR) signaling pathway to reduce the synthesis of protein in tissues, including the intestine [24].

The aim of this study was to investigate whether glycine could mitigate lipopolysaccharide (LPS)-induced intestinal injury, and to explore its molecular mechanism(s). We hypothesized that dietary glycine addition could enhance energy status and protein synthesis by suppressing AMPK activation and activating the mTOR signaling pathway, and reduce the production of pro-inflammatory cytokines in the intestine through regulating inflammatory signaling pathways to maintain intestinal integrity. LPS, a component of Gram-negative bacteria, is responsible for neonatal mortality and sepsis, but low concentrations of LPS resulted in tissue protection in some studies [25]. In the present study, *Escherichia coli* (*E. coli*) lipopolysaccharide (LPS; *E. coli* serotype 055:B5; potency ≥ 5,000,000 EU/mg) was intraperitoneally injected at 100 μg/kg BW (body weight), aimed to establish the model of endotoxemia [26]. Furthermore, we used the weanling piglet model, which is a suitable animal model for human nutrition research [27,28].

## 2. Results

### 2.1. Growth Performance

During the 28 days feeding period (before LPS or saline injection), there was no difference in average daily gain (510 ± 38 g), average feed intake (785 ± 76 g), and feed/gain ratio (1.54 ± 0.08) among the four groups.

### 2.2. Intestinal Morphology

**Figure 1:**
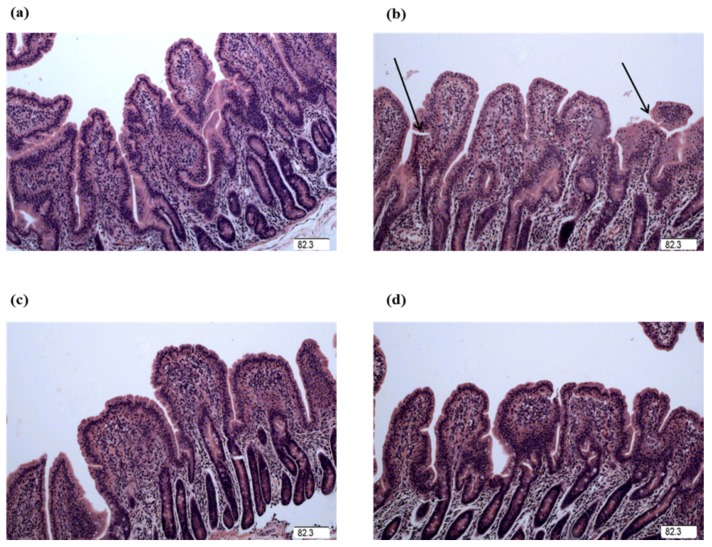
Jejunal mucosal histological appearance (hematoxylin and eosin). (**a**) Pigs fed a control diet and injected with sterile saline. No obvious damage was observed. (**b**) Pigs fed a control diet and injected with LPS. Intestinal mucosa was seriously damaged by LPS. Arrow represents the damaged intestinal mucosa in the piglets. (**c**) Pigs fed a 1.0% Gly diet and injected with LPS. Intestinal damage was alleviated. (**d**) Pigs fed a 2.0% Gly diet and injected with LPS. Intestinal damage was alleviated. Original magnification 100×. Scale bars = 82.3 μm.

**Figure 2:**
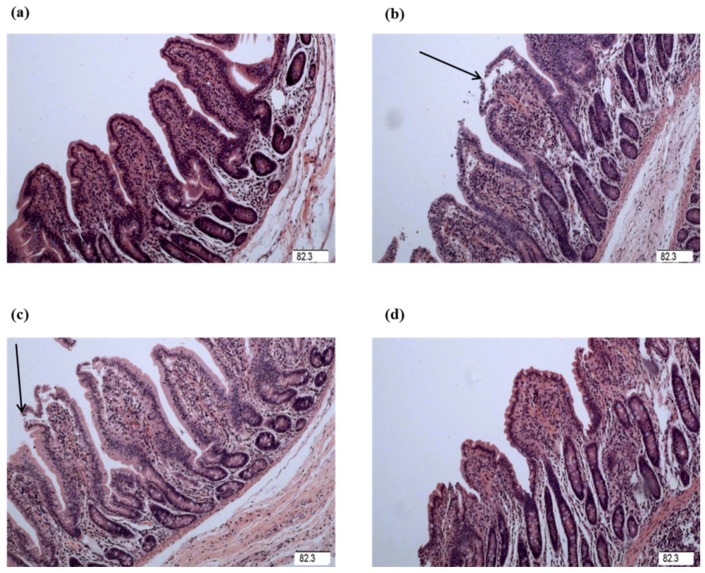
Ileal mucosal histological appearance (hematoxylin and eosin). (**a**) Pigs fed a control diet and injected with sterile saline. No obvious damage was observed. (**b**) Pigs fed a control diet and injected with LPS. Intestinal mucosa was seriously damaged by LPS. Arrow represents the damaged intestinal mucosa in the piglets. (**c**) Pigs fed a 1.0% Gly diet and injected with LPS. Intestinal damage was still existed. (**d**) Pigs fed a 2.0% Gly diet and injected with LPS. Intestinal damage was alleviated. Arrow represents the damaged intestinal mucosa in the piglets. Original magnification 100×. Scale bars = 82.3 μm.

The intestinal mucosa in control group was in good condition (Figure 1 and Figure 2). However, piglets challenged with LPS exhibited intestinal mucosal damage. Supplementation with Gly alleviated intestinal mucosal injury, to some extent. Compared to the CONTR (control group) piglets, the intestinal morphology in LPS-challenged piglets had no significant difference (*p* > 0.05). Among the LPS-challenged piglets, glycine supplementation decreased jejunal crypt depth (linear, *p* < 0.05; Table 1), and increased jejunal villus height/crypt depth ratio (VCR; linear, *p* < 0.05; quadratic, *p* < 0.05). However, there was no significant effect on intestinal morphology among the LPS-challenged piglets fed diets supplemented with glycine (*p* > 0.05).

### 2.3. Mucosal Protein, DNA, and RNA Content

Among the LPS-challenged piglets, supplementation with glycine increased mucosal protein content (linear, *p* < 0.05; Table 2), RNA/DNA ratio (linear, *p* < 0.05), and protein/DNA ratio (linear, *p* < 0.05) in jejunum. Compared with piglets in the CONTR group, the piglets in LPS group had a reduced RNA/DNA ratio in the ileum (*p* < 0.05). Furthermore, the piglets challenged with LPS had increased mucosal protein content (linear, *p* < 0.05; quadratic, *p* < 0.05), and RNA/DNA ratio (linear, *p* < 0.05) in the ileum, when they were fed diets supplemented with glycine.

### 2.4. Intestinal Claudin-1 Protein Expression

**Figure 3:**
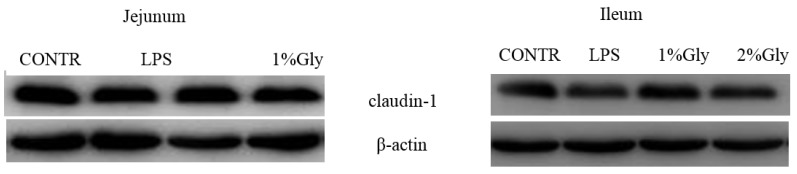
Effects of glycine supplementation on intestinal protein abundance of claudin-1 after 4 h LPS challenge in piglets.

As shown in Table 3 and Figure 3, the piglets challenged with LPS had decreased claudin-1 protein abundance in the ileum, compared with the piglets in CONTR group (*p* < 0.05). However, there was no significant difference in claudin-1 protein abundance in the jejunum between CONTR and LPS group (*p* > 0.05). In addition, there was no significant effect on the intestinal claudin-1 protein abundance in the piglets fed diets supplemented with glycine (*p* > 0.05).

### 2.5. Intestinal Key Enzyme Activities of the Tricarboxylic Acid (TCA) Cycle

Compared with the piglets in CONTR group, the piglets in LPS group had decreased ileal citrate synthase (CS) and α-ketoglutarate dehydrogenase complex (α-KGDHC) activities (*p* < 0.05; Table 4). Among the piglets challenged with LPS, supplementation with glycine in the diets increased the activities of α-KGDHC (quadratic, *p* < 0.05) in jejunum, and the activity of CS (quadratic, *p* < 0.05) in ileum.

### 2.6. Intestinal Protein Expression of the Key Protein in AMPKα and mTOR Pathways

**Figure 4:**
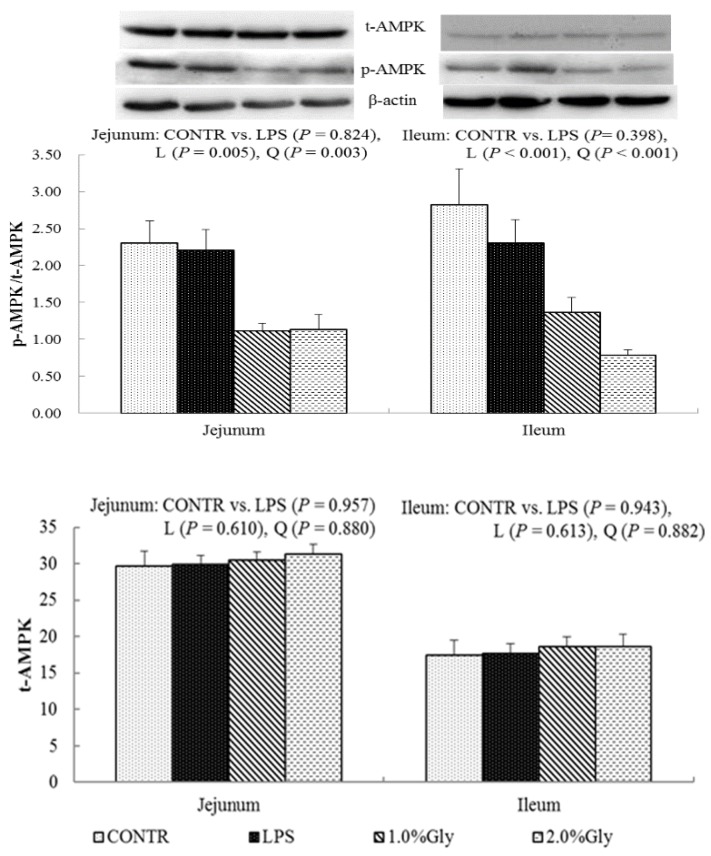
Effects of glycine supplementation on intestinal protein abundance of t-AMPKα and p-AMPKα after 4 h LPS challenge in weanling piglets. Note: L (linear), Q (quadratic).

**Figure 5:**
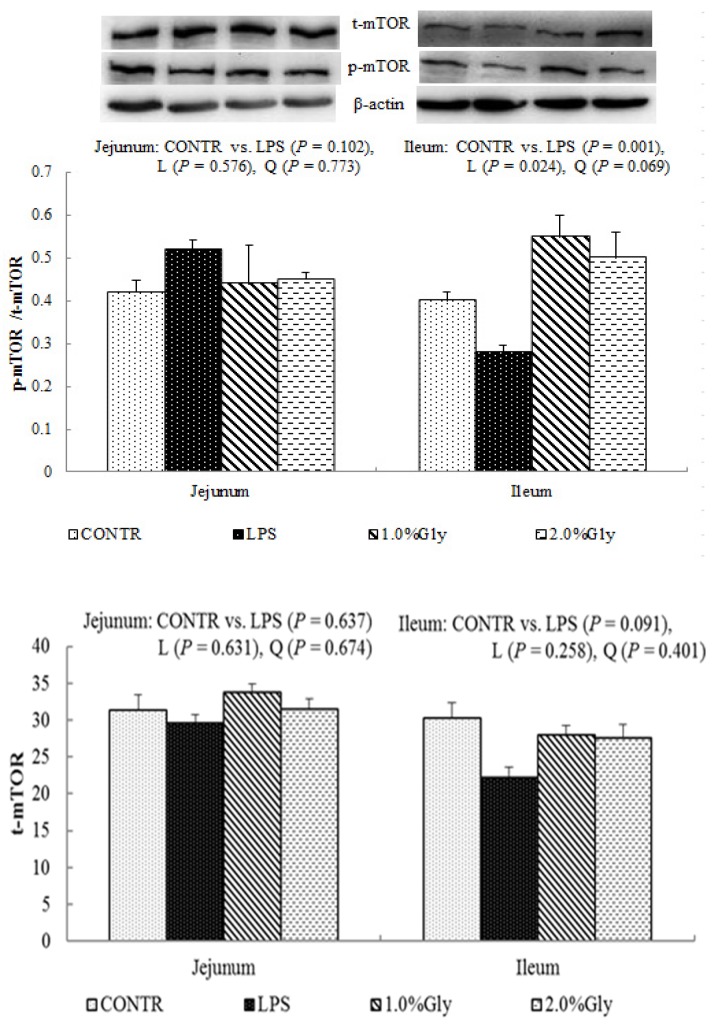
Effects of glycine supplementation on intestinal protein abundance of t-mTOR and p-mTOR after 4 h LPS challenge in weanling piglets. Note: L (linear), Q (quadratic).

**Figure 6:**
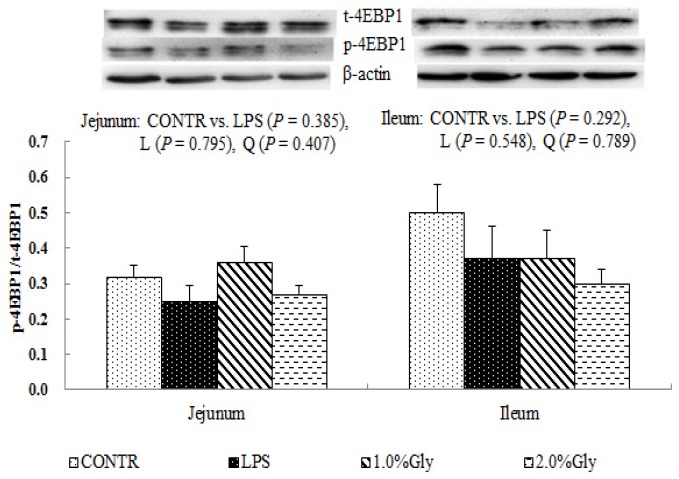

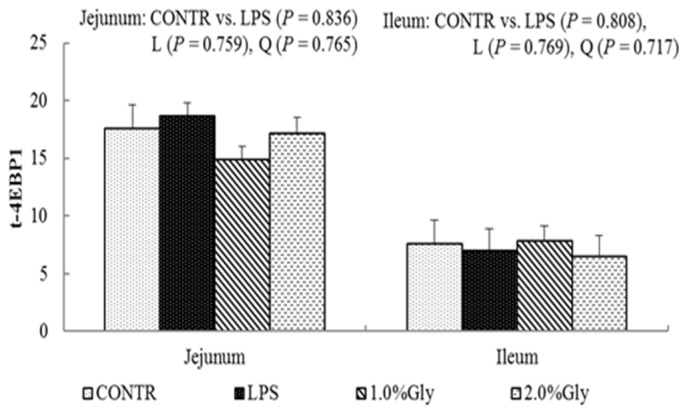
Effects of glycine supplementation on intestinal protein abundance of t-4EBP1 and p-4EBP1 after 4 h LPS challenge in weanling piglets. Note: L (linear), Q (quadratic).

Among the LPS-challenged piglets, supplementation with glycine decreased the ratio of p-AMPKα/t-AMPKα both in jejunum and ileum (linear, *p* < 0.05; quadratic, *p* < 0.05; Figure 4). Relative to CONTR piglets, LPS challenge decreased the ratio of p-mTOR/t-mTOR in ileum (*p* < 0.05; Figure 5), while there was no difference in the ratio of p-mTOR/t-mTOR in jejunum between CONTR and LPS group (*p* > 0.05). As supplementation with glycine in the diets, the piglets challenged with LPS increased ratio of p-mTOR/t-mTOR in ileum (linear, *p* < 0.05). However, there was no effect of glycine or LPS on the ratio of t-AMPKα, t-mTOR, p-4EBP1, and t-4EBP1 in jejunum and ileum (*p* > 0.05; Figure 4, Figure 5 and Figure 6).

### 2.7. Intestinal mRNA Expression of the Key Genes in TLR4 and NOD Pathways

Relative to CONTR piglets, the LPS pigs had higher mRNA expression of TLR4, MyD88, NOD2, RIPK2, and NF-κB (*p* < 0.05; Table 5) in jejunum. Among LPS challenged piglets, supplementation with glycine decreased mRNA expression of TLR4, LBP, MyD88, TRAF6, NOD2, and NF-κB (linear, *p* < 0.05; quadratic, *p* < 0.05) in jejunum. Compared to the piglets in CONTR group, the piglets in LPS group had increased mRNA expression of NOD2, and RIPK2 (*p* < 0.05) in ileum. However, the mRNA expression of IRAK1 in piglets challenged with LPS was reduced compared with the piglets in CONTR group (*p* < 0.05). Glycine supplementation reduced mRNA expression of NOD2 and RIPK2 (quadratic, *p* < 0.05) in ileum among the LPS-challenged pigs.

### 2.8. Intestinal mRNA Expression of Negative Regulators of TLR4 and NOD Pathways

Compared with the pigs in CONTR group, LPS challenge decreased jejunal mRNA expression of Tollip (*p* < 0.05; Table 6), and ileal mRNA expression of Tollip, ERBB2IP, and centaurin β1 (*p* < 0.05) in the piglets. However, LPS challenge increased mRNA expression of SOCS1 in jejunum (*p* < 0.05) and SIGIRR in ileum (*p* < 0.05). Among the LPS challenged piglets, supplementation with glycine increased mRNA expression of Tollip (quadratic, *p* < 0.05) and ERBB2IP (linear, *p* < 0.05) in ileum.

## 3. Discussion

In the present study, the effect of glycine on intestinal integrity after a 4 h *E. coli* LPS challenge was evaluated in a weaned piglet model. Dietary glycine supplementation improved the intestinal energy status and protein synthesis associated with inhibiting AMPK signaling and activating mTOR signaling, and simultaneously reduced the intestinal inflammatory response associated with inhibiting inflammatory signaling pathways (TLR4 and NOD), and as a consequence, improved intestinal integrity.

The intestinal mucosal integrity of weanling piglets is closely related to physical health and nutrient digestion and absorption capability [24,29]. Villus height, crypt depth, and VCR were determined to indicate gross intestinal morphology [3]. In this study, the decreased jejunal crypt depth and increased jejunal VCR of the piglets fed glycine indicated that the digestive juice can be secreted easily into intestinal lumen, which showed glycine played a role in maintaining the integrity in mucosal structure in weanling piglets. Wang et al. reported supplementation with glycine increased intestinal villus height in nursery piglets [7]. Effenberger-Neidnicht et al. found that supplementation with glycine improved intestinal architecture and reduced the LPS-induced intestinal accumulation of blood in rats [11]. The above studies indicated that glycine had a positive effect on the intestinal mucosal integrity.

Protein and DNA are the basis for repair and proliferation of epithelial cells. The ratio of RNA/DNA is an indicator reflecting the cell capacity for protein synthesis [30]. The ratio of protein/DNA is a sensitive indicator of protein mass and cell size [24]. The decreased ileal RNA/DNA ratio in LPS-challenged piglets indicated that the mucosal cell capacity for repair was decreased, affected by LPS. The piglets fed diets containing glycine had increased jejunal and ileal protein mass, protein/DNA ratio, and RNA/DNA ratio in the present study. These results illustrated that glycine is beneficial to intestine mucosal repair after LPS-induced injury. Similar to our data, Lee et al. [31] reported that glycine supplementation increased intestinal protein mass in the rats under ischemia–reperfusion injury. In addition, Stoll et al. [32] demonstrated that glycine could be utilized directly to synthesize protein in the intestinal tract. Furthermore, Wang et al. [7] demonstrated that glycine was the precursor of purines, which were involved in protein synthesis and cell proliferation.

The intestinal epithelial barrier can reduce the ability of luminal pathogens and their toxins to invade the mucosa, preventing the penetration of luminal bacteria into the mucosa, to maintain gut homeostasis [33]. The disordered function of the intestinal epithelial barrier often leads to inflammatory disease in the intestine [34]. The integrity of the intestinal epithelial barrier is maintained by cohesive interactions between epithelial cells to form tight junctions [34]. Claudin-1, which is an important protein in formation of tight junctions, determines permeability characteristics in many tissues, especially the intestine [35]. Therefore, the greater abundance of claudin-1 often reflects improved function of the epithelial barrier [36]. In the present study, LPS injection decreased the ileal protein abundance of claudin-1, which showed that LPS impaired the function of intestinal epithelial barrier. Similarly, some previous reports demonstrated that LPS challenge decreased the abundance of claudin-1 [37]. However, supplementation with glycine in the diets did not affect the abundance of claudin-1 in the piglets in our study. Few reports studied the effect of glycine on the intestinal tight junctions, so the pathway to repair injured tight junction in the intestine should be further studied in the future.

Citrate synthase, ICD, and α-KGDHC are key enzymes involved in the TCA cycle, which is a central route for energy production [38]. CS catalyzes the first step of the TCA cycle by attaching molecules of acetate and attaching them to oxaloacetate [39]. ICD, which exists in mitochondria and cytoplasm, is responsible for catalyzing the oxidative decarboxylation of isocitrate into α-ketoglutarate and CO_2_ [40]. α-KGDHC is a multi-enzymatic complex which converts α-ketoglutarate into succinyl-CoA [41]. In our experiment, LPS challenge reduced the activities of ICD in jejunum and CS, and α-KGDHC in ileum, which indicated that the energy production efficiency decreased. This is consistent with a previous report which showed that LPS challenge decreased the activities of jejunal CS, ICD, and α-KGDHC, and ileal ICD in weanling piglets [4]. After supplementation with glycine in the diets, the activities of α-KGDHC in jejunum and CS in ileum were increased. Therefore, it is possible that glycine could improve the energy production efficiency in intestinal mucosa by enhancing the key enzyme activities of the TCA cycle.

We hypothesized that glycine had a beneficial effect on intestinal integrity through enhancing energy production and protein synthesis in epithelial cells. AMPK, as an energy regulator, maintains the intracellular energy balance in eukaryons [42]. When the intracellular AMP/ATP ratio increases, AMPK is activated by phosphorylation [43]. The activated AMPK can switch on ATP-producing processes while synchronously switching off ATP-consuming processes to restore the cellular energy status [44]. Supplementation with glycine decreased jejunal and ileal p-AMPK/t-AMPK ratio, which illustrated dietary glycine could potentially enhance ATP-consumption to synthesize protein. mTOR is a serine–threonine kinase which controls many important aspects in mammalian cell functions, such as protein synthesis [45]. Its activity is modulated by various intracellular and extracellular factors (especially AAs and energy), meanwhile, mTOR adjusts rates of translation, transcription, protein degradation, cell signaling, and metabolism [44]. Specially, mTOR signaling plays a critical role in maintaining intestinal health [45,46]. Similar to our results, a previous report showed that LPS injection decreased jejunal mRNA expression of mTOR [47]. It is known that LPS could inhibit intestinal protein synthesis through suppressing activation of mTOR. The piglets fed diets supplemented with glycine had increased ileal p-mTOR/t-mTOR ratio, which demonstrated glycine could relieve the reduction of mucosal protein synthesis caused by LPS injury in the ileum. These results are in agreement with the decreased p-AMPK/t-AMPK ratio in piglets fed the diets supplemented with glycine. Therefore, we propose that the mechanism may be that glycine improves the intestinal mucosal energy status, and further activates mTOR signaling pathway to enhance protein synthesis and mucosal repair.

We hypothesized that supplementation with glycine improved intestinal integrity by inhibiting intestinal TLR4 and NODs, and their respective downstream signals, further reducing the inflammatory response. In the current study, LPS injection increased the mRNA abundance of TLR4 (TLR4, MyD88, and NF-κB in jejunum) and NOD signaling-related genes (NOD2 and RIPK2 in jejunum and ileum) which reflected LPS-induced intestinal inflammation by activation of TLR4 and NOD pathways. Supplementation with glycine reduced mRNA expressions of TLR4 (TLR4, LBP, MyD88, TRAF6, and NF-κB in jejunum) and NOD signaling-related genes (NOD2 and RIPK2 in jejunum and ileum) in the LPS-challenged piglets, which illustrated that glycine could relieve intestinal inflammation by inhibiting activation of inflammatory signaling pathways. In general, the inflammatory cytokines, such as TNF-α, IL-1β, and IL-6 can be overproduced when the inflammatory signaling pathways (TLR4 and NODs) are activated. Similar to our study, Tsune et al. [10] reported that glycine relieved colitis in rats by reducing mRNA expression of TNF-α and IL-1β. Furthermore, Stoffels et al. [48] demonstrated that injection of glycine before intestinal surgery could alleviate inflammation by decreasing mRNA expression of IL-6 and TNF-α.

Activation of TLR4 and NOD signaling could prevent against pathogens invading by triggering the production of pro-inflammatory cytokines and inflammatory response. However, over activation of inflammatory signaling pathways also lead to collateral host tissue injury [49]. To prevent excessive and harmful inflammatory responses, these inflammatory signaling pathways are negatively controlled by multiple mechanisms. So far, many negative regulators of TLR4 signaling (Tollip and SOCS1) and NOD signaling (ERBB2IP and centaurin β1) have been identified and characterized [50,51]. The current results showed that the piglets challenged with LPS had decreased mRNA expression of Tollip both in jejunum and ileum, as well as ERBB2IP and centaurin β1 in ileum, which is in agreement with the report of Wang et al. [52]. These results reflect that LPS challenge reduced the mRNA expression of TLR4 and NOD negative regulators, which is in agreement with increased mRNA expression of TLR4 and NOD signaling-related genes. Fujimoto et al. reported that excessive inflammatory cytokines enforced the expression of SOCS1, which resulted in decreased response of cells to TLR ligands [53]. In accordance with this, our present study showed that LPS challenge increased mRNA expressions of jejunal SOCS1, which indicates that SOCS1 might play a key role in intestinal self-protection. Supplementation with glycine increased mRNA expression of ileal ERBB2IP. Previous studies showed that Tollip bound to IL-1 receptor-associated kinase (IRAK) and inhibited IRAK phosphorylation to downregulate TLR4 signaling [54]. This indicates that supplementation with glycine could suppress the activity of IRAK by increasing the mRNA expression of Tollip, resulting in impairing the signaling from TLR4 to downstream pathways, and reducing the synthesis of pro-inflammatory cytokines.

In the present study, the effects of LPS challenge or glycine supplementation on some parameters were inconsistent in different sites of the intestine. This may be due to the difference in the anatomy and physiology among the different sites of the intestine [37]. In addition, LPS caused dynamic changes in the physiological variables, and gene and protein expression of inflammatory signaling pathways [55,56]. After 28 days feeding of Gly, the efficacy of Gly has risen. However, having only one time point (4 h) selected, to measure the effect of LPS on various physiological variables and gene and protein expression, was not perfect. Therefore, in future studies, sample collections at more time points are needed to better understand the dynamic effect of LPS on intestinal injury. Furthermore, the maintenance of normal blood flow through microcirculation plays a fundamental role in the protection and healing of intestinal mucosa [57]. Many previous studies reported that the intestinal injury induced by various factors can be alleviated through improving blood flow in microcirculation of duodenum and colon [58,59,60,61]. In the future, the blood flow of jejunal and ileal mucosa needs to be measured to better explore the mechanism of effects of LPS or glycine on the intestine.

## 4. Materials and Methods

### 4.1. Animal Care and Diets

The experiments and animal care were approved by Animal Care and Use Committee of Wuhan Polytechnic University, Hubei Province, China (EM628, 18 June 2016). Twenty-four weanling crossbred barrows (Duroc × (Landrace × Large White), 28 ± 1 days of age), with an average body weight (BW) of 7.17 ± 0.41 kg were used in this study. Piglets were housed individually in stainless steel metabolic cages (1.80 × 1.10 m^2^). All piglets had free access to feed and water in an environmentally controlled house, with ambient temperature maintained at 22–25 °C. The piglets were getting routine immunization, and in good health condition without fever or diarrhea. The living environment was in accordance with animal welfare guidelines in the whole experimental period. The basal diet (Table 7) met or exceeded the National Research Council requirements for all nutrients [62].

### 4.2. Experimental Design

All piglets were randomly divided into 4 treatments: (1) non-challenged control (CONTR; piglets fed a basal diet and injected with 0.9% NaCl solution); (2) LPS-challenged control (LPS; piglets fed the same basal diet and injected with *E. coli* LPS (*E. coli* serotype 055:B5; potency ≥ 5,000,000 EU/mg; Sigma Chemical, St. Louis, MO, USA)); (3) LPS + 1.0% glycine group (piglets fed a 1.0% glycine-supplemented diet and injected with LPS); and (4) LPS + 2.0% glycine group (piglets fed a 2.0% glycine-supplemented diet and injected with LPS). We selected a supplementary dose of glycine in accordance with a previous study by Wang et al. [7]. In order to get isonitrogenous diets, 2.38%, 1.19%, and 0% alanine (purity > 99%; Amino Acid Bio-Chemical Co., Wuhan, China) were supplemented to the control, 1.0% glycine, and 2.0% glycine diets, respectively. After a 28 days feeding period, the piglets in the control, 1.0% glycine, and 2.0% glycine groups were given an intraperitoneal injection of LPS at 100 μg/kg BW or the same volume of 0.9% NaCl solution. After administration with LPS or NaCl solution, all piglets were deprived of feed for 4 h until slaughter, so as to avoid the potential influence on the intestinal mucosa caused by feed intake change [63].

### 4.3. Intestinal Sample Collections

Four hours after injection with saline or LPS, all piglets were euthanized via injection of sodium pentobarbital. A 3 cm and 10 cm segments were collected from each mid-jejunum and mid-ileum, referred to in our previous study [64]. Many previous studies demonstrated that LPS resulted in intestinal morphologic impairment and dysfunction within 3–6 h postinjection, and it is caused by increased production of pro-inflammatory cytokines [3,64,65]. Therefore, we choose the time point of 4 h following saline or LPS administration for sample collection. The 3 cm intestinal segments were gently flushed and stored in 4% paraformaldehyde/PBS until histological analysis [3,64]. The 10 cm intestinal samples were opened longitudinally, and gently flushed to remove digesta. The mucosa samples were collected by scraping using sterile glass slides, then rapidly frozen in liquid nitrogen and stored at −80 °C until measuring of DNA, RNA, protein content, tricarboxylic acid (TCA) cycle key enzyme activities, mRNA, and protein expression levels. The intestinal samples were collected within 15 min after slaughter.

### 4.4. Intestinal Morphology Analysis

The 3 cm intestinal segment samples were cut into small pieces, not exceeding 2 mm, and enclosed into plastic tissue cassettes then processed over a 19 h period in an automatic tissue processor. Fixed intestinal samples were prepared according to the conventional paraffin-embedding techniques [66]. Samples were cut to 5 μm thickness and then stained with hematoxylin and eosin. Villus height and crypt depth were determined at 40× magnification using a microscope (Olympus CX31, Tokyo, Japan). Ten well-oriented and intact villi were selected at least. Villus height was defined from the tip of the villus to the villus–crypt junction, and crypt depth was obtained as the depth of the invagination between adjacent villi [3].

### 4.5. Intestinal Mucosal Protein, DNA, and RNA Contents

Frozen mucosal samples were firstly ground using a pestle with supplementation of liquid nitrogen, and then homogenized in ice-cold saline water at a 1:10 (*w*/*v*) ratio, then centrifuged at 2500 rpm for 10 min (4 °C) to collect the supernatant. The supernatant was used to measure protein, RNA, and DNA contents. The content of intestinal mucosal protein was measured referred to the method of Zhu et al. [67]. The content of DNA was measured through a fluorometric assay [68]. The content of RNA was determined through spectrophotometry described by Schmidt-Tannhauser [69].

### 4.6. Intestinal Mucosal TCA Cycle Key Enzyme Activities

The key enzymes involved in TCA cycle include citrate synthase (CS), isocitrate dehydrogenase (ICD), and α-ketoglutarate dehydrogenase complex (α-KGDHC). The activities of these enzymes in the intestinal mucosal supernatant were determined according to the previous report by Wang et al. [4], and assayed via commercial enzyme assay kits (#45126 for CS, #45234 for ICD and #45157 for α-KGDHC; Shanghai Yuanye Biotechnology Company, Shanghai, China).

### 4.7. mRNA Abundance Analysis

All the procedures, such as RNA extraction, quantification, reverse transcription, as well as real-time PCR, were carried out according to a previous report [65]. Primer pairs used in the present study are shown in Table 8. The relative expression of target genes to housekeeping gene (GAPDH) was analyzed via the 2^−ΔΔ*C*t^ method [70]. The results showed that the gene expression of GAPDH was similar among 4 treatments. Relative mRNA expression of the target gene was normalized to the control group (piglets fed a control diet and injected with 0.9% NaCl solution). In detail, the 2^−ΔΔ*C*t^ method is also named the comparative cycle threshold (*C*_t_) method, where C_T_ is the number of cycles required to reach an arbitrary threshold. The *C*_t_ for target gene of each sample was corrected by subtracting the *C*_t_ for GAPDH (Δ*C*_t_). The jejunal and ileal segments of the CONTR group were chosen as reference samples, and the Δ*C*_t_ for all experimental samples was subtracted by the average Δ*C*_T_ for the reference samples (ΔΔ*C*_t_). Finally, experimental mRNA abundance relative to control mRNA abundance was calculated with use of the formula 2^−ΔΔ*C*t^. We tested several housekeeping genes by analyzing gene stability as described by Vandesompele et al. [71]. We found the expression of GAPDH to be more stable than that of other housekeeping genes, and there was no variation in the expression of GAPDH among intestinal segments and treatments.

### 4.8. Protein Abundance Analysis

Protein abundance of claudin-1, AMPK, mTOR, and 4EBP1 in intestinal mucosa was measured in accordance with a previous study [66]. In brief, the intestinal mucosa samples (150–200 mg) were homogenized in 600 μL lysis buffer containing phenylmethanesulfonylfluoride, protease, and phosphatase inhibitors, and centrifuged at 12,000× *g* for 15 min at 4 °C to collect supernatants. Equal amounts of intestinal mucosa protein (65 μg) were transferred onto 10–15% polyacrylamide gel and separated via SDS-PAGE, and then transferred to polyvinylidene difluoride membranes for immunoblotting. Immunoblots were blocked with 5% nonfat milk in Tris-buffered saline/Tween-20 for 3 h at room temperature (21–25 °C). The membranes were incubated overnight at 4°C with primary antibodies, and then with the secondary antibodies for 2 h at room temperature. Specific primary antibodies were used, including rabbit anti-claudin-1 (1:1000) (No. 519000, Invitrogen Technology Inc., Danvers, MA, USA), rabbit anti-total AMPKα (t-AMPKα, 1:1000) (No. 2532, Cell Signalling Technology Inc., Danvers, MA, USA), rabbit anti-phosphorylated AMPKα (p-AMPKα, 1:1000) (No. 2535, Cell Signalling Technology Inc.), rabbit anti-total mTOR (t-mTOR, 1:1000) (No. 2972, Cell Signalling Technology Inc.), rabbit anti-phosphorylated mTOR (p-mTOR, 1:1000) (No. 2971, Cell Signalling Technology Inc.), rabbit anti-total eukaryotic initiation factor 4E binding protein 1 (t-4EBP1, 1:1000) (No. 9452, Cell Signalling Technology Inc.), rabbit anti-phosphorylated 4EBP1 (p-4EBP1, 1:1000) (No. 9455, Cell Signalling Technology Inc. and mouse anti-β-actin (1:10,000) (No. A2228, Sigma Aldrich Inc., St. Louis, MO, USA). The secondary antibodies included goat anti-rabbit IgG-HRP (1:5000) (No. ANT019, Antgene Biotech Inc., Wuhan, China), and rabbit anti-goat IgG-HRP (1:5000) (No. ANT020, Antgene Biotech Inc.). Blots were developed using an Enhanced Chemiluminescence Western blotting kit (Amersham Biosciences, Solna, Sweden), and visualized using a Gene Genome bioimaging system. Bands were analyzed by densitometry using Gene Tool software (Syngene, Frederick, MD, USA). β-Actin was used as a loading control in Western blotting analysis. The relative protein abundance of claudin-1 was expressed as claudin-1/β-actin. Phosphorylated form of AMPKα, mTOR and 4EBP1 were normalized with the total protein content.

### 4.9. Statistical Analysis

All data were analyzed as a randomized complete block design using the mixed procedure (SAS Inst. Inc., Cary, NC, USA). Individual piglets were used as the experimental unit for all statistic procedures. The data were firstly adjusted by homogeneity of variance. The model included treatment as main effect, and replicates as random effects. The treatment effects were tested using the following contrasts: (1) CONTR vs. LPS was used to test the effect of LPS challenge; (2) The different dose–response effects of glycine were tested using linear and quadratic trends for the three glycine levels (0, 1.0, and 2.0% glycine) among piglets challenged with LPS. Results were shown as mean and pooled SEM. Significant differences were declared at *p* < 0.05.

## 5. Conclusions

In summary, supplementation with glycine improves intestinal integrity in piglets after LPS challenge. The beneficial effects of glycine on the intestine may be related to (1) enhancing mucosal energy status and protein synthesis via maintaining mTOR and inhibiting AMPK signaling; (2) decreasing intestinal inflammation through inhibiting TLR4 and NOD signaling pathways.

## Figures and Tables

**Table 1:** Effect of glycine supplementation on intestinal morphology after 4 h lipopolysaccharide (LPS) challenge in piglets.

Item	Treatment ^1^				SEM	*p* Value ^2^		
	CONTR	LPS	LPS + 1.0% Gly	LPS + 2.0% Gly		CONTR vs. LPS	Linear	Quadratic
Jejunum								
Villus height (μm)	286	286	268	265	9.7	0.973	0.111	0.201
Crypt depth (μm)	106	103	88.6	88.1	5.0	0.688	0.037	0.058
VCR	2.76	2.82	2.91	2.94	0.03	0.356	0.012	0.038
Ileum								
Villus height (μm)	244	241	255	254	10.0	0.902	0.368	0.560
Crypt depth (μm)	83.0	81.0	85.9	86.2	3.5	0.758	0.230	0.412
VCR	2.95	2.93	2.97	2.94	0.03	0.740	0.739	0.467

^1^ CONTR (non-challenged control), piglets fed a basal diet as well as injected with 0.9% NaCl solution; LPS (LPS-challenged control), piglets fed the same basal diet as well as injected with *E. coli* LPS; LPS + 1.0% Gly, piglets fed a 1.0% glycine-supplemented diet and injected with LPS; LPS + 2.0% Gly, piglets fed a 2.0% glycine-supplemented diet and injected with LPS. VCR, villus height/crypt depth ratio. SEM, standard error of mean. ^2^ CONTR vs. LPS was used to obtain the response of LPS challenge. Linear and quadratic polynomial contrasts were used to obtain the response of glycine supplementation in LPS-challenged piglets.

**Table 2:** Effect of glycine supplementation on intestinal protein, DNA and RNA contents after 4 h LPS challenge in piglets.

Item	Treatment ^1^				SEM	*p* Value ^2^		
	CONTR	LPS	LPS + 1.0% Gly	LPS + 2.0% Gly		CONTR vs. LPS	Linear	Quadratic
Jejunum								
Protein (mg/g tissue)	84.4	81.3	89.7	90.8	3.7	0.614	0.049	0.099
RNA/DNA	3.30	3.35	3.77	3.82	0.20	0.888	0.049	0.097
Protein/DNA (mg/μg)	0.20	0.18	0.21	0.21	0.01	0.365	0.021	0.055
Ileum								
Protein (mg/g tissue)	56.3	61.7	68.8	72.3	2.3	0.231	<0.001	0.001
RNA/DNA	11.4	8.29	9.64	9.65	0.59	0.011	0.048	0.125
Protein/DNA (mg/μg)	0.31	0.27	0.32	0.34	0.03	0.395	0.069	0.180

^1^ CONTR (non-challenged control), piglets fed a basal diet as well as injected with 0.9% NaCl solution; LPS (LPS-challenged control), piglets fed the same basal diet as well as injected with *E. coli* LPS; LPS + 1.0% Gly, piglets fed a 1.0% glycine-supplemented diet and injected with LPS; LPS + 2.0% Gly, piglets fed a 2.0% glycine-supplemented diet and injected with LPS. SEM, standard error of mean. ^2^ CONTR vs. LPS was used to obtain the response of LPS challenge. Linear and quadratic polynomial contrasts were used to obtain the response of glycine supplementation in LPS-challenged piglets.

**Table 3:** Effects of glycine supplementation on intestinal protein abundance of claudin-1 after 4 h LPS challenge in piglets.

Item	Treatment ^1^				SEM	*p* Value ^2^		
	CONTR	LPS	LPS + 1.0% Gly	LPS + 2.0% Gly		CONTR vs. LPS	Linear	Quadratic
Jejunum								
claudin-1/β-actin	0.43	0.50	0.52	0.47	0.07	0.282	0.772	0.902
Ileum								
claudin-1/β-actin	1.03	0.60	0.80	0.64	0.13	0.032	0.814	0.482

^1^ CONTR (non-challenged control), piglets fed a basal diet as well as injected with 0.9% NaCl solution; LPS (LPS-challenged control), piglets fed the same basal diet as well as injected with *E. coli* LPS; LPS + 1.0% Gly, piglets fed a 1.0% glycine-supplemented diet and injected with LPS; LPS + 2.0% Gly, piglets fed a 2.0% glycine-supplemented diet and injected with LPS. SEM, standard error of mean. ^2^ CONTR vs. LPS was used to obtain the response of LPS challenge. Linear and quadratic polynomial contrasts were used to obtain the response of glycine supplementation in LPS-challenged piglets.

**Table 4:** Effects of glycine supplementation on intestinal tricarboxylic acid cycle key enzyme activities after 4 h LPS challenge in weaned piglets.

Item	Treatment ^1^				SEM	*p* Value ^2^		
	CONTR	LPS	LPS + 1.0% Gly	LPS + 2.0% Gly		CONTR vs. LPS	Linear	Quadratic
Jejunum								
CS (U·g protein^−1^)	1.88	1.66	1.68	1.57	0.10	0.255	0.388	0.560
ICD (mIU·g protein^−1^)	187	168	173	190	7.82	0.088	0.064	0.708
α-KGDHC (μg·g protein^−1^)	3146	2706	2171	2844	169	0.140	0.615	0.015
Ileum								
CS (U·g protein^−1^)	1.37	1.07	0.93	1.21	0.05	0.002	0.192	0.015
ICD (mIU·g protein^−1^)	127	109	110	108	7.62	0.160	0.941	0.979
α-KGDHC (μg·g protein^−1^)	2566	1638	1783	1966	182	0.019	0.196	0.443

CS: citrate synthase, ICD: isocitrate dehydrogenase, α-KGDHC: α-ketoglutarate dehydrogenase complex. SEM, standard error of mean. ^1^ CONTR (non-challenged control), piglets fed a basal diet as well as injected with 0.9% NaCl solution; LPS (LPS-challenged control), piglets fed the same basal diet as well as injected with *E. coli* LPS; LPS + 1.0% Gly, piglets fed a 1.0% glycine-supplemented diet and injected with LPS; LPS + 2.0% Gly, piglets fed a 2.0% glycine-supplemented diet and injected with LPS. ^2^ CONTR vs. LPS was used to obtain the response of LPS challenge. Linear and quadratic polynomial contrasts were used to obtain the response of glycine supplementation in LPS-challenged piglets.

**Table 5:** Effect of glycine supplementation on intestinal mRNA expression of TLR4 and NOD and their downstream signals after 4 h LPS challenge in piglets.

Item	Treatment ^1^				SEM	*p* Value ^2^		
	CONTR	LPS	LPS + 1.0% Gly	LPS + 2.0% Gly		CONTR vs. LPS	Linear	Quadratic
Jejunum								
TLR4	1.00	1.67	1.17	1.22	0.09	<0.001	0.005	0.001
LBP	1.00	0.82	0.67	0.44	0.11	0.439	0.005	0.022
MyD88	1.00	1.17	0.92	0.93	0.04	0.017	0.007	0.003
IRAK1	1.00	1.00	0.89	0.95	0.06	0.943	0.579	0.509
TRAF6	1.00	1.22	0.84	0.93	0.07	0.079	0.034	0.007
NOD1	1.00	0.66	0.59	0.53	0.11	0.103	0.249	0.524
NOD2	1.00	2.45	1.42	1.53	0.20	0.006	0.019	0.011
RIPK2	1.00	1.84	1.39	1.68	0.09	<0.001	0.331	0.010
NF-κB	1.00	1.29	1.02	1.10	0.05	0.001	0.039	0.006
Ileum								
TLR4	1.00	1.13	1.22	1.20	0.08	0.215	0.557	0.713
LBP	1.00	0.44	0.27	0.33	0.21	0.198	0.418	0.474
MyD88	1.00	1.04	0.95	0.98	0.06	0.646	0.403	0.431
IRAK1	1.00	0.83	0.69	0.92	0.08	0.021	0.490	0.199
TRAF6	1.00	1.15	1.07	1.16	0.06	0.125	0.972	0.583
NOD1	1.00	0.86	0.81	0.85	0.10	0.415	0.926	0.890
NOD2	1.00	1.97	1.07	1.54	0.20	0.006	0.255	0.039
RIPK2	1.00	1.85	1.37	1.82	0.11	<0.001	0.900	0.019
NF-κB	1.00	1.08	1.02	1.09	0.06	0.381	0.886	0.729

IRAK1: IL-1 receptor-associated kinase, LBP: lipopolysaccharide-binding protein, MyD88: myeloid differentiation factor 88, NF-κB: nuclear factor-κB, NOD1: nucleotide-binding oligomerization domain protein 1, NOD2: nucleotide-binding oligomerization domain protein 2, RIPK2: receptor-interacting protein kinase 2, TLR4: toll-like receptor 4, TRAF6: TNF receptor-associated factor 6, SEM: standard error of mean. ^1^ CONTR (non-challenged control), piglets fed a basal diet as well as injected with 0.9% NaCl solution; LPS (LPS-challenged control), piglets fed the same basal diet as well as injected with *E. coli* LPS; LPS + 1.0% Gly, piglets fed a 1.0% glycine-supplemented diet and injected with LPS; LPS + 2.0% Gly, piglets fed a 2.0% glycine-supplemented diet and injected with LPS. ^2^ CONTR vs. LPS was used to obtain the response of LPS challenge. Linear and quadratic polynomial contrasts were used to obtain the response of glycine supplementation in LPS-challenged piglets.

**Table 6:** Effects of glycine supplementation on intestinal mRNA expression of negative regulators of TLR4 and NOD signal pathway after 4 h LPS challenge in piglets.

Item	Treatment ^1^				SEM	*p* Value ^2^		
	CONTR	LPS	LPS + 1.0% Gly	LPS + 2.0% Gly		CONTR vs. LPS	Linear	Quadratic
Jejunum								
RP105	1.00	1.94	0.95	1.59	0.34	0.190	0.558	0.216
SOCS1	1.00	2.74	1.69	1.94	0.30	0.019	0.128	0.103
Tollip	1.00	0.52	0.93	0.59	0.12	0.016	0.702	0.062
SIGIRR	1.00	0.96	1.01	1.00	0.05	0.524	0.525	0.736
ERBB2IP	1.00	0.88	0.76	0.79	0.04	0.074	0.136	0.125
centaurin β1	1.00	0.82	0.56	0.58	0.08	0.202	0.029	0.027
Ileum								
RP105	1.00	0.73	0.79	0.84	0.10	0.116	0.355	0.661
SOCS1	1.00	0.87	1.03	1.10	0.08	0.242	0.063	0.170
Tollip	1.00	0.62	1.16	0.64	0.12	0.007	0.942	0.008
SIGIRR	1.00	1.23	1.13	1.26	0.06	0.001	0.797	0.328
ERBB2IP	1.00	0.77	0.82	0.97	0.06	0.028	0.029	0.082
centaurin β1	1.00	0.74	0.75	0.80	0.07	0.041	0.431	0.696

ERBB2IP: Erbb2 interacting protein, RP105: radioprotective 105, SIGIRR: single immunoglobulin IL-1 related receptor, SOCS1: suppressor of cytokine signaling 1, Tollip: toll-interacting protein, SEM: standard error of mean. ^1^ CONTR (non-challenged control), piglets fed a basal diet, as well as injected with 0.9% NaCl solution; LPS (LPS-challenged control), piglets fed the same basal diet as well as injected with *E. coli* LPS; LPS + 1.0% Gly, piglets fed a 1.0% glycine-supplemented diet and injected with LPS; LPS + 2.0% Gly, piglets fed a 2.0% glycine-supplemented diet and injected with LPS. ^2^ CONTR vs. LPS was used to obtain the response of LPS challenge. Linear and quadratic polynomial contrasts were used to obtain the response of glycine supplementation in LPS-challenged piglets.

**Table 7:** Ingredient composition of experimental diets (%, as-fed basis).

Ingredients		Nutrient Level ^4^	
Corn	57.02	Digestible energy (MJ/Kg)	13.5
Soybean meal (44% CP)	21.40	Crude protein	18.7
Wheat middling	5.00	Crude fat	4.75
Fish meal	3.60	Ca	0.88
Soy protein concentrate	1.40	Total P	0.67
Fat powder	2.00	Lysine	1.02
Defatted milk-replacer powder	3.00	Methionine + Cystine	0.72
Limestone	0.94	Threonine	0.74
Dicalcium phosphate	1.22	Glycine	0.70
Salt	0.34		
Alanine ^1^	2.38		
l-Lysine HCl (78.8%)	0.27		
dl-Methionine (99%)	0.10		
l-Threonine (98%)	0.08		
Acidifier ^2^	0.20		
Butylated hydroquinone	0.05		
Vitamin and mineral premix ^3^	1.00		

^1^ In the 1.0% glycine diet, we used 1.0% glycine, 1.19% alanine and 0.19% cornstarch to replace 2.38% alanine. In the 2.0% glycine diet, we used 2.0% glycine and 0.38% cornstarch to replace 2.38% alanine. We made all diets isonitrogenous. ^2^ A compound acidifier (lactic acid and phosphoric acid), was purchased from Wuhan Fanhua Biotechnology Company, Wuhan, China. ^3^ Premix (defatted rice bran as carrier) supplied per kg diet: retinol acetate, 2700 μg; cholecalciferol, 62.5 μg; dl-α-tocopheryl acetate, 20 mg; menadione sodium bisulfite complex, 3 mg; riboflavin, 4 mg; d-calcium-pantothenate, 15 mg; niacin, 40 mg; choline chloride, 400 mg; folic acid, 700 μg; thiamin, 1.5 mg; pyridoxine, 3 mg; biotin, 100 μg; Mn (MnSO_4_·5H_2_O), 20 mg; Fe (FeSO_4_·H_2_O), 83 mg; Zn (ZnSO_4_·7H_2_O), 80 mg; Cu (CuSO_4_·5H_2_O), 25 mg; I (KI), 0.48 mg; Se (Na_2_SeO_3_·5H_2_O), 0.36 mg. ^4^ The nutrients level was analyzed value except digestible energy which is calculated value.

**Table 8:** Primer sequences used for real-time PCR.

Gene	Forward (5′-3′)	Reverse (5′-3′)	Product Length (bp)	Accession Numbers
*TLR4*	TCAGTTCTCACCTTCCTCCTG	GTTCATTCCTCACCCAGTCTTC	166	GQ503242.1
*LBP*	GAACACAGCCGAATGGTCTAC	GGAAGGAGTTGGTGGTCAGT	151	NM 001128435.1
*MyD88*	GATGGTAGCGGTTGTCTCTGAT	GATGCTGGGGAACTCTTTCTTC	148	AB292176.1
*IRAK1*	CAAGGCAGGTCAGGTTTCGT	TTCGTGGGGCGTGTAGTGT	115	XM_003135490.1
*TRAF6*	CAAGAGAATACCCAGTCGCACA	ATCCGAGACAAAGGGGAAGAA	122	NM_001105286.1
*RP105*	CGAGGCTTCTGACTGTTGTG	GGTGCTGATTGCTGGTGTC	245	AB190767.1
*SOCS1*	GCGTGTAGGATGGTAGCA	GAGGAGGAGGAGGAGGAAT	101	NM_001204768.1
*Tollip*	GCAGCAGCAACAGCAGAT	GGTCACGCCGTAGTTCTTC	133	AB490123.1
*SIGIRR*	ACCTTCACCTGCTCCATCCA	TTCCGTCATTCATCTCCACCTC	205	AB490122.1
*NOD1*	CTGTCGTCAACACCGATCCA	CCAGTTGGTGACGCAGCTT	57	AB187219.1
*NOD2*	GAGCGCATCCTCTTAACTTTCG	ACGCTCGTGATCCGTGAAC	66	AB195466.1
*RIPK2*	CAGTGTCCAGTAAATCGCAGTTG	CAGGCTTCCGTCATCTGGTT	206	XM_003355027.1
*NF-κB*	AGTACCCTGAGGCTATAACTCGC	TCCGCAATGGAGGAGAAGTC	133	EU399817.1
*ERBB2IP*	ACAATTCAGCGACAGAGTAGTG	TGACATCATTGGAGGAGTTCTTC	147	GU990777.1
*centaurin β1*	GAAGCCGAAGTGTCCGAATT	AGGTCACAGATGCCAAGAATG	125	XM_003358258.2
*GAPDH*	CGTCCCTGAGACACGATGGT	GCCTTGACTGTGCCGTGGAAT	194	AF017079.1

ERBB2IP: Erbb2 interacting protein, GAPDH: glyceraldehyde-3-phosphate dehydrogenase, IRAK1: IL-1 receptor-associated kinase, LBP: lipopolysaccharide-binding protein, MD2: myeloid differentiation protein 2, MyD88: myeloid differentiation factor 88, NF-κB: nuclear factor-κB, NOD1: nucleotide-binding oligomerization domain protein 1, NOD2: nucleotide-binding oligomerization domain protein 2, RIPK2: receptor-interacting protein kinase 2, RP105: radioprotective 105, SIGIRR: single immunoglobulin IL-1 related receptor, SOCS1: suppressor of cytokine signaling 1, TLR4: toll-like receptor 4, Tollip: toll-interacting protein, TRAF6: TNF receptor-associated factor 6.
